# Curve Sprint in Elite Female Soccer Players: Relationship with Linear Sprint and Jump Performance

**DOI:** 10.3390/ijerph18052306

**Published:** 2021-02-26

**Authors:** Ronaldo Kobal, Tomás T. Freitas, Alberto Fílter, Bernardo Requena, Renato Barroso, Marcelo Rossetti, Renato M. Jorge, Leonardo Carvalho, Lucas A. Pereira, Irineu Loturco

**Affiliations:** 1 Sport Club Corinthians Paulista, São Paulo 03828-000, Brazil; rokorin2002@hotmail.com (R.K.); marcelorosseti5@gmail.com (M.R.); renatomljorge@yahoo.com (R.M.J.); 2 School of Physical Education—University of Campinas, Campinas 13083-851, Brazil; rbarroso@unicamp.br (R.B.); leo_carvalho_1994@hotmail.com (L.C.); 3 NAR-Nucleus of High Performance in Sport, São Paulo 04753-060, Brazil; tfreitas@ucam.edu (T.T.F.); lucasa_pereira@outlook.com (L.A.P.); 4 Department of Human Movement Sciences—Federal University of São Paulo, São Paulo 11015-020, Brazil; 5 UCAM Research Center for High Performance Sport—Catholic University of Murcia, 30107 Murcia, Spain; 6 Football Science Institute (FSI), 18016 Granada, Spain; albertofr_91@hotmail.com (A.F.); bernardorequena@icloud.com (B.R.); 7 Faculty of Sport-University of Pablo de Olavide (UPO), 41013 Seville, Spain; 8 Faculty of Life Sciences and Education, University of South Wales, Pontypridd CF037 1DL, Wales, UK

**Keywords:** football, team sport, agility, sprinting, curved sprint

## Abstract

The aim of this study was to examine the associations between linear sprint, curve sprint (CS), change of direction (COD) speed, and jump performance in a sample of 17 professional female soccer players. All athletes performed squat and countermovement jumps, single leg horizontal triple jumps, 17 m linear sprints, CS tests, and a 17 m Zigzag COD test. A Pearson product–moment test was performed to determine the relationships among the assessed variables. The significance level was set at *p* < 0.05. Nearly perfect associations (*r* > 0.9) were found between linear and CS velocities. Players faster in linear sprints and CS exhibited greater COD deficits. No significant associations were found between COD deficit and either body mass or sprint momentum. Jumping ability was significantly correlated with linear sprint and CS performance, but not to COD performance. These findings may be used by coaches and practitioners to guide testing and training prescriptions in this population. The associations observed here suggest that training methods designed to improve linear sprint and CS velocities may benefit from the implementation of vertically and horizontally oriented plyometric exercises.

## 1. Introduction

The physical performance and match-play demands of elite men’s soccer players have been widely investigated [1,2]. However, information on top-level female players is scant, even though the sport is becoming increasingly popular. Different studies have shown that a women’s soccer match is characterized by intermittent efforts, with players covering ~10.3 km [3] and changing locomotor activity more than 1000 times per game (i.e., every ~4 s) [4]. Furthermore, data from competitions indicate that sprinting actions are frequent and determinant during match-play [3,4,5]. For example, Mohr et al. [4] reported that higher-level female players cover a greater distance (~24%) while sprinting than do their less skilled counterparts (i.e., lower-level players). Since the majority of sprint efforts in soccer are non-linear, this emphasizes the need to develop sprinting ability in this population, not only over straight courses but also along multidirectional trajectories [6,7,8].

Consequently, the study of multidirectional sprints in soccer (i.e., curvilinear or incorporating directional changes) and their association with other speed and power qualities has received great attention in recent years [7,9,10]. For instance, investigations of female soccer players have reported close relationships between the change of direction (COD) speed and linear sprint performance, vertical jump height, and lower-body power [9,10]. Moreover, it has also been shown that eccentric [11], isometric, and reactive strength [9] may be important determinants of COD performance. The literature is scarce with regard to curve sprint (CS), and no previous research has investigated this ability in women’s soccer, although it is a critical action, as 85% of the actions performed at maximum velocity are curvilinear [6]. Only two recent studies [12,13] have reported on moderate to large associations between CS outcomes and performance in the vertical jump, sprint, and COD, and both works were conducted with male soccer players. Therefore, there is a clear need to examine the capability of female athletes to perform sprinting actions over curvilinear trajectories.

The ability to apply and orient a larger quantity of horizontal forces onto the ground influences performance in sprint and COD [14,15,16]. For this reason, and due to their ease of application, horizontal jump tests are frequently included in soccer player testing protocols [17,18,19]. Nonetheless, the relationship between bilateral horizontal jump and sprint performances is somewhat unclear. On one hand, there is evidence of large associations between sprint speed and horizontal jump distances in soccer players [17,20]. On the other hand, McCurdy et al. [21] reported that horizontal jumps (i.e., bilateral and unilateral) were not correlated to either 10- or 25 m sprint times in NCAA Division I female soccer players. These contradictory results indicate that more research is warranted to better understand these interrelationships, especially in elite female soccer players.

The present study examines the associations between linear sprint, CS, COD, and jump (vertical and horizontal) performances in a sample of elite female soccer players. Based on previous findings regarding male soccer players, we hypothesized that linear, CS, COD, and jump performances would be positively correlated, with stronger relationships regarding horizontally oriented jumps.

## 2. Materials and Methods

### 2.1. Participants

Seventeen elite female soccer players (age: 25.6 ± 3.7 years; height: 165.7 ± 5.6 cm; body mass (BM): 60.7 ± 5.6 kg) from the same team participated in this study. Soccer players were assessed immediately before starting the preseason period. At the time of the study, the team was the *Libertadores da América Cup* champion and included 8 players who had competed at the highest level of soccer in Brazil, as members of the Brazilian National Soccer Team. Prior to study participation, all players signed an informed consent form. The study was approved by the Federal University of São Paulo Ethics Committee/(ethical approval code: 4.355.629).

### 2.2. Study Design

This cross-sectional correlational study was designed to determine the associations between linear sprint, CS, COD, and jump (vertical and horizontal) performances in elite players. On the same day, athletes had their BM measured, and then performed, in the same order: squat jump (SJ), countermovement jump (CMJ), single-leg horizontal triple jump (THJ), a 17 m linear sprint, a CS test, and a 17 m Zigzag COD test. All players were well familiarized with testing procedures due to their regular assessments in our facilities. Prior to the tests, players performed a standardized warm-up which included general (i.e., running at a moderate pace for 10 min followed by stretching exercises for 3 min) and specific exercises (i.e., submaximal jump and sprint attempts). Between each test, a 10 min interval was provided to explain the procedures, allow adequate recovery, and adjust the equipment.

### 2.3. Procedures

#### 2.3.1. Body Mass

BM was measured using a digital scale with an accuracy of 0.1 kg (Filizola Industry, São Paulo, Brazil). Measurements were performed prior to the warm-up activity, with the athletes barefoot and wearing training clothes.

#### 2.3.2. Vertical Jumping Tests

Vertical jump height was assessed for the SJ and CMJ. In the SJ, the players were required to remain in a static position with a 90° knee flexion angle for ~2 s before jumping, without any preparatory movement. In the CMJ, players started from a standing position and were instructed to execute a downward movement followed by a complete extension of the legs, and they were free to determine the countermovement depth to avoid changes in jumping coordination. All jumps were executed with the hands on the hips, and the players were instructed to jump as high as possible. The jumps were performed on a contact platform (Elite Jump^®^, S2 Sports, São Paulo, Brazil), and jump heights were calculated based on flight time [22]. Players were instructed to land in the same place as they took off. A total of five attempts were allowed for each jump, interspersed by 15 s intervals. The best attempts for the SJ and CMJ were used for subsequent analyses.

#### 2.3.3. Horizontal Jumping Tests

Athletes performed the THJ starting from a standing position. Arm swing was allowed to provide maximal forward drive. The test consisted of players performing three maximal horizontal unilateral jumps, in sequence, with the same leg. The jump distance, in cm, was determined using a metric tape measure (Lufkin L716MAGCME, Appex Group, Chicago, USA), from the take-off line to the nearest point of landing contact, measured from the back of the heel. Each athlete executed three attempts at each jump for each leg, interspersed by 30 s intervals, and the trial with the greatest distance was retained for the analysis. Data from the “good” (longest distance) and “weak” (shortest jump distance) legs were obtained from the best attempt of each side for each jump mode.

#### 2.3.4. Linear Sprint Test

Two pairs of photocells (Smart Speed, Fusion Sport, Brisbane, Australia) were positioned at the starting line and a distance of 17 m. The players sprinted twice, starting from a standing position 0.5 m behind the starting line. Sprint velocity (VEL 17 m) was calculated as the distance traveled over a measured time interval. A 5 min rest interval was allowed between the two attempts, and the fastest time was considered for analysis. Sprint momentum (SM, kg·m·s^−1^) was obtained by multiplying each athlete’s BM by their velocity during the linear sprint.

#### 2.3.5. Curve Sprint Test

**Figure 1:**
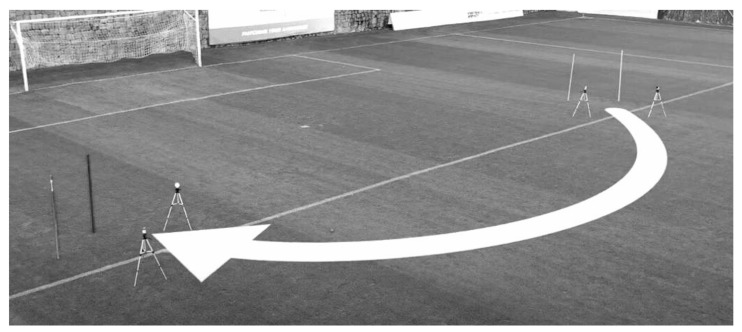
Schematic presentation of the curve sprint test.

The CS test was performed as previously described [7] (Figure 1). The trajectory of the CS was the semi-circle of the goalkeeper area of an official soccer field, which is standardized as follows: a 9.15 m radius from the penalty spot; a 14.6 m distance from the initial to the final point in a straight line; an angle of 105.84° amplitude from the point of the penalty spot; a 17 m total distance (obtained from a trigonometrical analysis). Two pairs of photocells (Smart Speed, Fusion Sport, Brisbane, Australia) were positioned at the beginning and the end of the curved trajectory. Soccer players sprinted two times for each side (first to the left and, subsequently, to the right), starting from a standing position 0.5 m behind the starting line. A 5 min rest interval was allowed between the two attempts for each side, and the fastest time was considered for analysis. From the best attempt of each side, the “good side” (fastest time, CSGS) and the “weak side” (slowest time, CSWS) were recorded. To properly assess player efficiency in using linear speed during a CS, a CS deficit calculation was used for both the good and weak sides. The CS deficit was calculated as follows: “CS_DEF_ = (17 m sprint velocity–CS velocity)/CS velocity × 100”.

#### 2.3.6. Modified Zigzag Change of Direction Test

The modified Zigzag COD test consisted of four 4.25 m sections (total 17 m of linear distance) marked with cones set at 100° angles [13,23] requiring the athletes to decelerate and accelerate as fast as possible around each cone. Two maximal attempts were performed, with a 5 min rest interval between attempts. Starting from a standing position 0.5 m behind the first pair of timing gates (Smart Speed, Fusion Equipment, Brisbane, Australia), athletes were instructed to complete the test as quickly as possible, until crossing the second pair of timing gates, placed 17 m from the starting line. The fastest time from the two attempts was retained. To evaluate the efficiency of players using their linear speed during a COD maneuver, an adapted COD deficit calculation was used as follows: “COD_DEF_ = (17 m sprint velocity–COD velocity)/COD velocity × 100”.

### 2.4. Statistical Analysis

The normality of the data was confirmed using the Shapiro–Wilk test. Data are presented as mean, standard deviation, and 95% confidence limits. A Pearson product–moment test was performed to determine the relationships among the distinct variables tested. The correlation coefficients were qualitatively interpreted as follows: <0.09, trivial; 0.10–0.29, small; 0.30–0.49, moderate; 0.50–0.69, large; 0.70–0.89, very large; >0.90 nearly perfect [23]. The significance level was set at *p* < 0.05. All physical tests performed demonstrated high levels of absolute and relative reliability (i.e., intraclass correlation coefficients >0.90 and coefficients of variation <5%) [24].

## 3. Results

**Figure 2:**
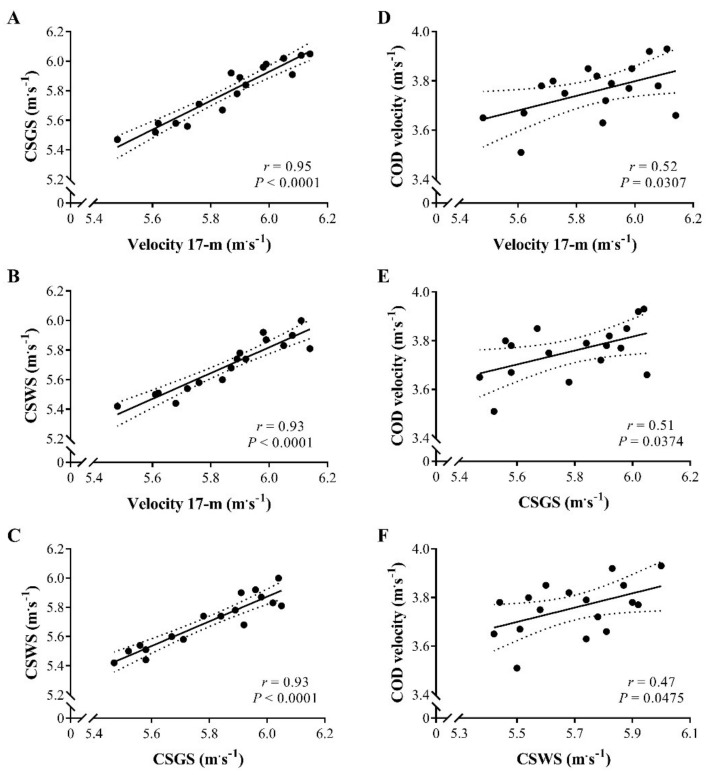
(**A**–**F**) Linear regressions between 17 m linear sprint velocity, curve sprint velocity for the good and weak sides (CSGS and CSWS, respectively), and change of direction (COD) velocity.

Table 1 shows the descriptive data of the physical tests performed with the soccer players. Figure 2 depicts the linear regressions between the players’ linear, curve, and COD velocities. Nearly perfect significant relationships were shown between the linear and curve sprint for both sides, and also between the good and weak sides of the curve sprint. Moderate to large significant correlation coefficients were noticed between the COD velocity and the linear and curve sprint performances.

Table 2 shows the correlation coefficients among the linear (i.e., VEL 17 m) and multidirectional velocities (i.e., CSGS, CSWS, and COD), the BM and SM, and the deficits (i.e., CSGS_DEF_, CSWS_DEF_, and COD_DEF_). Significant and large associations were found between COD_DEF_ and VEL 17 m, CSGS, and CSWS. Table 3 displays the relationships between the linear and multidirectional velocities and the jump performances. SJ and CMJ were significantly correlated with VEL 17 m, CSGS and CSWS to a moderate-to-large degree. Moderate to large and significant associations were obtained between THJ with both legs and the distinct linear and multidirectional sprints.

## 4. Discussion

The aim of this study was to investigate the relationships between linear sprint, CS, COD, and jump (vertical, and horizontal) performances in elite female soccer players. The main findings indicated that (1) nearly perfect associations (*r* > 0.9) were found between linear and CS velocities; (2) players faster in linear and CS displayed greater COD_DEF_; however, no significant associations were found between COD_DEF_ and BM or SM; and (3) jumping ability was significantly correlated with linear sprint and CS performance but not with COD performance.

The associations presented between linear sprint and CS velocities for both the players’ good and weak sides are the strongest reported in the literature to date (*r* = 0.93–0.95). Previous studies [12,13] also detected strong correlations ranging from 0.74 to 0.82 in young male soccer players. The nearly perfect relationships observed herein may suggest that higher-level players (i.e., professional, and national team athletes) are able to better express and utilize their linear sprint capacity over curvilinear paths, possibly through an optimized action of the inside leg (considered the main limiting factor when sprinting over curved trajectories) and a superior ability to cope with high centripetal forces [13,25,26,27,28]. In a sample of semi-professional soccer players, Filter et al. [26] demonstrated that the inside and outside legs present different behaviors and kinematic patterns during CS actions. The inside leg displays higher contact times and resembles a “continuum cross-step maneuver,” while the outside leg displays shorter ground contact times and exhibits an action similar to a “continuum side-step maneuver” [26]. From this information, it is plausible to assume that the inter-leg differences (i.e., inside vs. outside leg) between the linear sprint and CS actions were less marked in the female players assessed in this study than those assessed in other investigations [12,13]. Additional studies involving a greater number of team-sport athletes are required before more robust conclusions can be drawn.

The kinematic adjustments observed during CS may contribute to the moderate to large associations with COD velocity found here and elsewhere [12]. The whole-body movement strategies adopted by athletes are similar when sprinting over curved paths or when performing shallow cutting maneuvers (e.g., body lateral inward lean or thigh separation of the legs at touchdown) [25]. These biomechanical similarities may partially explain the significant relationships obtained, despite the fact that the braking requirements in the Zigzag maneuver were considerably higher than they were during the CS test (in which velocity maintenance was key) [29]. From an applied perspective, CS and shallow–moderate COD seem to share some common technical and physical elements. Hence, training strategies aimed at developing skills such as side-step cutting and drills promoting the application of mediolateral ground reaction forces of high magnitude might positively influence CS performance in female soccer players.

The CSGS_DEF_ and CSWS_DEF_ were not significantly related to any of the variables tested. The COD_DEF_ was largely associated with linear sprint velocity but not to the BM or SM. Notably, a recent investigation analyzing COD ability, sprinting speed, and momentum in elite female athletes from different team sports found similar results concerning soccer players [30], indicating that linear velocity appears to be the most influential variable contributing to lower efficiency when changing direction (i.e., higher COD_DEF_) in this population. This seems not to be the case for male soccer players [31,32] or for players of other sports such as rugby [30,33] in which athletes typically present a considerably heavier BM, and SM plays a more determinant role by being relatively higher by providing greater momentum for the same absolute velocity. One possible explanation for this phenomenon may be that the combination of linear velocity and BM is not a problematic issue for female soccer players, as the ability to change direction is more dependent on training background or other physical and technical factors (e.g., strength–power capacity, leg muscle characteristics, foot placement, and body position) [34]. In practical terms, it can be inferred that training programs focused on improving the ability to tolerate greater entry velocities [29] such as eccentric-based training [35,36], technique-oriented tasks (e.g., working on proper body positioning or foot placement, etc.), or acceleration–deceleration drills [37] may be more effective than strategies targeted at optimizing body composition (e.g., reducing body fat) in an attempt to decrease SM.

The large relationships found between the vertical jump height and sprint velocities were not surprising in light of previously published data [10,13,38,39,40,41]. As has been widely described, the association between these variables may be explained by the fact that jumping and sprinting both require the application of considerable amounts of vertical force onto the ground to rapidly accelerate the body vertically or forward [13,42,43,44]. Interestingly, our findings reinforce the conclusions from two recent studies concerning CS outcomes in young male soccer players [12,13] and provide the first evidence that female athletes with superior vertical jump ability tend to be faster in linear and curved sprinting actions. The close association of horizontal jump performance with linear sprint and CS is noteworthy. During the acceleration phase, the ability to orient the resultant force vector horizontally is a determinant factor for sprint performance [15], and it is also the case for the horizontal jump distance. Hence, the relationships observed were somewhat expected [45] and support the notion that complement vertical plyometrics with horizontally oriented jumping exercises is an effective strategy to improve sprint ability in team-sport athletes [46]. Overall, jumping variables were not associated with COD velocity, with the exception of THJG. In fact, COD is a multifaceted quality in which multiple physical and technical aspects (aside from force production can influence performance [14,47]. We speculate that when performing consecutive unilateral jumps with the “good” leg, a combination of factors ranging from greater propulsive and braking force application to superior trunk or hip control explain why the THJG distance was the only variable associated with COD velocity [48,49]. More research is warranted to better understand these interrelationships considering, in particular, asymmetries between the dominant and non-dominant legs.

The present study is limited by its cross-sectional design that precludes any causal inferences. Likewise, the facts that the sample comprised only professional players and that a single COD test (i.e., Zigzag) was performed should be considered; therefore, our findings cannot be extrapolated to athletes from other competitive levels or different COD maneuvers (e.g., pro-agility shuttle, 505, and *t*-test). Future studies should investigate whether multidirectional training strategies combining vertically and horizontally oriented speed–power exercises are effective in improving linear and CS performance, COD speed, and jumping ability in female soccer players.

## 5. Conclusions

The results of this study revealed for the first time the existence of (1) nearly perfect associations between linear and CS velocities and (2) moderate to large correlations between COD, linear, and CS abilities in elite female soccer players. Players who were faster in the linear and curved sprinting actions also exhibited greater COD_DEF_ and superior vertical and horizontal jump abilities. These findings may be used by coaches and practitioners to guide testing and training prescription in this specialized population. From a practical perspective, the associations obtained here indicate that training approaches designed to improve linear and CS velocities could benefit from the incorporation of vertically and horizontally oriented plyometric exercises. Furthermore, exercise programs focused on developing the ability to perform directional changes of approximately 90° may translate into an improved capability to sprint over curvilinear paths due to the mechanical similarities between these actions.

## Figures and Tables

**Table 1:** Descriptive data of the tested variables.

	Mean ± SD	95% CL	
		Lower	Upper
BM (kg)	60.7 ± 5.6	58.1	63.4
SJ (cm)	35.1 ± 2.7	33.8	36.3
CMJ (cm)	36.4 ± 2.6	35.2	37.7
THJG (cm)	559.0 ± 43.6	538.3	579.7
THJW (cm)	543.8 ± 40.7	524.4	563.1
VEL 17 m (m·s^−1^)	5.86 ± 0.19	5.77	5.95
SM (kg·m·s^−1^)	354.9 ± 26.6	342.2	367.5
CSGS (m·s^−1^)	5.79 ± 0.20	5.70	5.89
CSWS (m·s^−1^)	5.70 ± 0.18	5.61	5.78
COD VEL (m·s^−1^)	3.76 ± 0.11	3.71	3.81
CSGS_DEF_ (%)	1.20 ± 1.10	0.68	1.72
CSWS_DEF_ (%)	2.90 ± 1.23	2.32	3.49
COD_DEF_ (%)	56.0 ± 4.9	53.7	58.4

BM: body mass; CL: confidence limits; CMJ: countermovement jump; COD: change of direction; CSGS: curve sprint for the good side; CSWS: curve sprint for the weak side; DEF: deficit; SD: standard deviation; SJ: squat jump; SM: sprint momentum; THJG: triple horizontal jump with the good leg; THJW: triple horizontal jump with the weak leg; VEL: velocity.

**Table 2:** Correlation coefficients between linear and curve sprint and change of direction velocities, body mass, sprint momentum, and curve sprint and change of direction deficits.

	BM	VEL 17 m	SM	CSGS	CSWS	COD VEL	CSGS_DEF_	CSWS_DEF_
**CSGS_DEF_**	−0.24	−0.01	−0.38	−0.32	−0.16	−0.06		
**CSWS_DEF_**	−0.35	0.26	−0.34	0.12	−0.12	0.17	0.40	
**COD_DEF_**	−0.37	0.57 *	−0.16	0.52 *	0.53 *	−0.41	0.06	0.12

BM: body mass; COD: change of direction; CSGS: curve sprint for the good side; CSWS: curve sprint for the weak side; DEF: deficit; SM: sprint momentum; VEL: velocity. * *p* < 0.05.

**Table 3:** Correlation coefficients between jump performances, linear sprint, curve sprint, and change of direction velocities, and curve sprint and change of direction deficits.

	VEL 17 m	CSGS	CSWS	COD VEL	CSGS_DEF_	CSWS_DEF_	COD_DEF_
**SJ**	0.55 *	0.64 *	0.59 *	0.32	−0.40	−0.22	−0.30
**CMJ**	0.45 *	0.56 *	0.53 *	0.21	−0.43	−0.31	0.31
**THJG**	0.50 *	0.47 *	0.44 *	0.49 *	0.01	0.18	0.07
**THJW**	0.55 *	0.55 *	0.53 *	0.42	−0.05	0.02	0.21

CMJ: countermovement jump; COD: change of direction; CSGS: curve sprint for the good side; CSWS: curve sprint for the weak side; DEF: deficit; SJ: squat jump; THJG: triple horizontal jump with the good leg; THJW: triple horizontal jump with the weak leg VEL: velocity. * *p* < 0.05.

## Data Availability

All relevant data are within the manuscript.
